# Restrictive *versus* Liberal Fluid Therapy for Post-Cesarean Acute Kidney Injury in Severe Preeclampsia: a Pilot Randomized Clinical Trial

**DOI:** 10.6061/clinics/2020/e1797

**Published:** 2020-07-15

**Authors:** Wallace Andrino da Silva, Carlo Victor A. Varela, Aline Macedo Pinheiro, Paula Castro Scherer, Rossana P.V. Francisco, Marcelo Luis Abramides Torres, Maria José C. Carmona, Fernando Bliacheriene, Lúcia C. Andrade, Paolo Pelosi, Luiz Marcelo S. Malbouisson

**Affiliations:** I Departamento de Anestesia, Hospital das Clinicas HCFMUSP, Faculdade de Medicina, Universidade de Sao Paulo, Sao Paulo, SP, BR; II Departamento de Obstetricia e Ginecologia, Hospital das Clinicas HCFMUSP, Faculdade de Medicina, Universidade de Sao Paulo, Sao Paulo, SP, BR; III Departamento de Nefrologia, Hospital das Clinicas HCFMUSP, Faculdade de Medicina, Universidade de Sao Paulo, Sao Paulo, SP, BR; IV Dipartimento di Scienze Chirurgiche e Diagnostiche Integrate (DISC), Universitè degli Studi di Genova, Genoa, Italy; V IRCCS Ospedale Policlinico San Martino, Genoa, Italy

**Keywords:** Preeclampsia, Acute Kidney Injury, Cesarean Section, Fluid Therapy

## Abstract

**OBJECTIVES:**

The aim of this study was to determine whether a restrictive compared to a liberal fluid therapy will increase postoperative acute kidney injury (AKI) in patients with severe preeclampsia.

**METHODS:**

A total of 46 patients (mean age, 32 years; standard deviation, 6.8 years) with severe preeclampsia were randomized to liberal (1500 ml of lactated Ringer’s, n=23) or restrictive (250 ml of lactated Ringer’s, n=23) intravenous fluid regimen during cesarean section. The primary outcome was the development of a postoperative renal dysfunction defined by AKI Network stage ≥1. Serum cystatin C and neutrophil gelatinase-associated lipocalin (NGAL) were evaluated at postoperative days 1 and 2. ClinicalTrials.gov: NCT02214186.

**RESULTS:**

The rate of postoperative AKI was 43.5% in the liberal fluid group and 43.5% in the restrictive fluid group (*p=*1.0). Intraoperative urine output was higher in the liberal (116 ml/h, IQR 69-191) than in the restrictive fluid group (80 ml/h, IQR 37-110, *p*<0.05). In both groups, serum cystatin C did not change from postoperative day 1 compared to the preoperative period and significantly decreased on postoperative day 2 compared to postoperative day 1 (*p*<0.05). In the restrictive fluid group, NGAL levels increased on postoperative day 1 compared to the preoperative period (*p*<0.05) and decreased on postoperative day 2 compared to postoperative day 1 (*p*<0.05).

**CONCLUSION:**

Among patients with severe preeclampsia, a restrictive fluid regimen during cesarean section was not associated with increased postoperative AKI.

## INTRODUCTION

Preeclampsia is a multifactorial syndrome, clinically characterized by hypertension and proteinuria after 20 weeks of pregnancy. It occurs in 3–14% of pregnancies and is responsible for 60,000 maternal deaths worldwide each year. In patients with severe preeclampsia, the risk of pulmonary edema, coagulopathy, hemorrhage, and acute kidney injury (AKI) is higher (1). Since 2013, proteinuria is not an essential criteria for the diagnosis of preeclampsia, if any of these severe features are presented: thrombocytopenia, renal insufficiency, impaired liver function, pulmonary edema, and cerebral or visual symptoms (2).

In developed countries, the incidence of AKI has increased in the latest decades, especially among women with preeclampsia (3). In Canada, the incidence of AKI during pregnancy increased significantly from 1.66 to 2.68 per 10,000 deliveries between 2002 and 2010 (3), whereas it increased from 2.3 to 4.5 per 10,000 deliveries between 1998 and 2008 in the United States (4). In patients with severe preeclampsia undergoing cesarean section, the incidence of AKI has been reported up to 60%, but varying according to the type of patients, definition of AKI, and intraoperative management (5).

Fluid management in severe preeclampsia is controversial, especially fluid replacement during cesarean section. In severe preeclampsia, increased pulmonary capillary permeability and increased left ventricular end-diastolic pressure can lead to acute pulmonary edema even after intravenous volume replacement (6). Therefore, fluid restriction might prevent pulmonary edema (3,4,7). On the other hand, hypovolemia associated with fluid restriction can induce renal hypoperfusion and exacerbate AKI (3).

Although creatinine is a traditional marker of kidney function, serum creatinine increases only after ≥50% of renal function has been lost (8). In preeclampsia, early biomarkers of kidney injury have been studied to enable rapid diagnosis and early treatment for the preservation of kidney function (9-11). Cystatin C is a low-molecular-weight protein secreted by nucleated cells, freely filtered by the glomeruli, and completely reabsorbed/metabolized by the proximal tubules (12). Neutrophil gelatinase-associated lipocalin (NGAL) accumulates in blood within the first 3 hours after renal injury and remains elevated for several days after the initial insult (13).

Since the most effective intravenous fluid regimen is unclear, we conducted the present pilot trial to compare a restrictive fluid regimen with a more traditional (liberal) regimen in patients with severe preeclampsia during cesarean section surgery. Our primary hypothesis was that a restrictive fluid regimen would lead to a higher rate of postoperative AKI than a liberal fluid regimen in patients with severe preeclampsia who underwent cesarean section.

## MATERIALS AND METHODS

We performed a prospective randomized clinical trial. We evaluated all patients with severe preeclampsia admitted to the Obstetrics Department of the University of São Paulo School Of Medicine, Hospital das Clínicas with an indication for cesarean section between July 2014 and September 2015. The study was registered in the ClinicalTrials.gov database (identifier: NCT02214186; July 8, 2014) and was approved by the Hospital das Clínicas Committee for the Analysis of Research Projects (Ruling no. 01945012.6.0000.0068).

We included patients with severe preeclampsia undergoing cesarean section under spinal anesthesia. Patients were classified as having severe preeclampsia if they met at least one of the following criteria: systolic pressure at rest ≥160 mmHg or diastolic pressure at rest ≥110 mmHg on two occasions at least 4 hours apart (unless antihypertensive therapy was previously initiated); thrombocytopenia (platelet count <100 cells × 10^3^/mm^3^); impaired liver function (serum transaminases at least twice the normal concentration); severe persistent right upper quadrant or epigastric pain unresponsive to medication and not attributable to alternative diagnoses; pulmonary edema; and new-onset cerebral or visual disturbances. Although renal insufficiency is now considered a criterion of severity by the American College of Obstetricians and Gynecologists (2), it was not considered in this study because the occurrence of AKI was the primary outcome. Patients with a preoperative serum creatinine level >1.0 mg/dl were excluded, as were those with a history of renal disease, those undergoing another surgical procedure simultaneously, those receiving anesthesia other than spinal anesthesia, and those in whom labor had previously been induced. Beside this, those who did not accept to participate in the research and did not sign the informed consent form were excluded.

### Randomization and Intervention

All eligible patients were included in the randomization procedure. Allocation numbers were derived from a random number table and placed in envelopes that were opened only by the anesthesiologist who performed the spinal anesthesia. Patients were randomly allocated to a restrictive (250 ml of lactated Ringer’s) or liberal (1500 ml of lactated Ringer’s) fluid regimen during cesarean section. Informed consent was obtained immediately before the cesarean section.

To avoid any interference with the renal function, the use of nonsteroidal anti-inflammatory drugs and omeprazole was prohibited during hospitalization. Intraoperatively, patients received metaraminol (200 µg, intravenously) if their systolic blood pressure dropped by >20% in relation to the baseline value, with a subsequent bolus if the hypotension persisted or hydralazine (5 mg, intravenously) every 15 minutes if their blood pressure was >180/110 mmHg. Spinal anesthesia was performed in accordance with the institutional protocol—0.5% hyperbaric bupivacaine (15 mg), fentanyl (10 µg), and morphine (100 µg). No preloading was performed before spinal anesthesia. Postoperatively, all of the patients received an infusion of 10 units of oxytocin diluted in 500 ml of 5% dextrose over a 6-hour period, also in accordance with the institutional protocol, together with intramuscular administration of magnesium sulfate, as in the Pritchard regimen. None of the patients required additional maintenance of crystalloid infusion, but only the amount needed for drugs administration. Unrestricted diet and water ingestion were allowed for all patients 8 hours after cesarean section.

### Outcome Measures

The primary outcome measure was the change in serum creatinine on the first and second postoperative days compared to the preoperative values (baseline), used as a proxy for renal function. Using the modified Acute Kidney Injury Network (AKIN) classification (14), we stratified renal dysfunction into three categories, on the basis of the increase in serum creatinine over the preoperative value: stage 1 (150-200%), stage 2 (200-300%), and stage 3 (>300%). Patients requiring hemodialysis were also categorized as AKIN stage 3.

The secondary outcome measures were the serum levels of cystatin C and NGAL, proteinuria, platelet count, and intraoperative urine output.

The glomerular filtration rate (GFR) was estimated by the Modification of Diet in Renal Disease (MDRD) formula (15), as well as by a formula based on serum cystatin C and serum creatinine (16).

All these variables were measured at three time points: preoperative (baseline), on postoperative day 1 (PO1), and on postoperative day 2 (PO2). The variables were compared between the PO1 and baseline, PO2 and PO1, and then the PO2 and baseline.

Other intraoperative and postoperative variables that were not previously defined as secondary outcomes were presented as post-hoc analysis in the Appendix section. They were the intensive care unit (ICU) admission, vasopressor use, vasodilator use, supplemental oxygen use (oxygen saturation <94%), length of hospital stay, and umbilical cord blood gases from venous blood (pH, bicarbonate, and base excess), together with the 1- and 5-minute Apgar scores.

### Blinding and Data Quality

The attending anesthesiologists who conducted the cesarean section and the early postoperative care had knowledge of the group assignments, but were not included in the present research group. One of researchers staff followed the intraoperative and postoperative period to ensure that there was no breach in the study protocol. The research investigator responsible for analysing the outcome was blinded to the group assignment.

### Statistical Analysis

All the analyses were performed in a modified intention-to-treat population, which included all randomized patients with severe preeclampsia undergoing to cesarean section. All the patients were followed-up from the cesarean section until the hospital discharge.

The sample size selected was based on previously published studies. A recent population-based study performed by Mehrabadi et al. showed that hypertensive disorders were presented in 65.9% of the cases of obstetric acute renal failure (3). Huang et al. demonstrated that 60% of women with HELLP syndrome were complicated with AKI (5). Further, previous observations in our center suggested an AKI incidence of 60 to 80% in patients with severe features of preeclampsia. We must point out that variable incidence of postoperative AKI has been reported in the literature. This variation is due to the different types of patients enrolled (chronic hypertension, preeclampsia, severe preeclampsia, HELP syndrome, and eclampsia), methods to define AKI, and intraoperative management strategies among the different studies. Thus, assuming a power of 80% and a 95% confidence interval, the minimum sample necessary to conduct the study was calculated to be 46 patients (23 in each group of interest).

Quantitative variables were evaluated for normality with the Kolmogorov-Smirnov test. Variables with normal distribution were described as means and standard deviations, being compared by repeated-measures analysis of variance. Variables without normal distribution were described as medians and interquartile ranges, being compared by the nonparametric Kruskal-Wallis and Friedman tests. The Mann-Whitney test and Student’s *t*-test were used in comparisons of independent samples. Qualitative variables were described as absolute and relative frequencies, the chi-square test or Fisher’s exact test being used in determining associations. Values of *p*≤0.05 were considered significant. Statistical analyses were performed using the Statistical Package for the Social Sciences version 18.0 (SPSS Inc., Chicago, IL, USA).

## RESULTS

**Figure 1:**
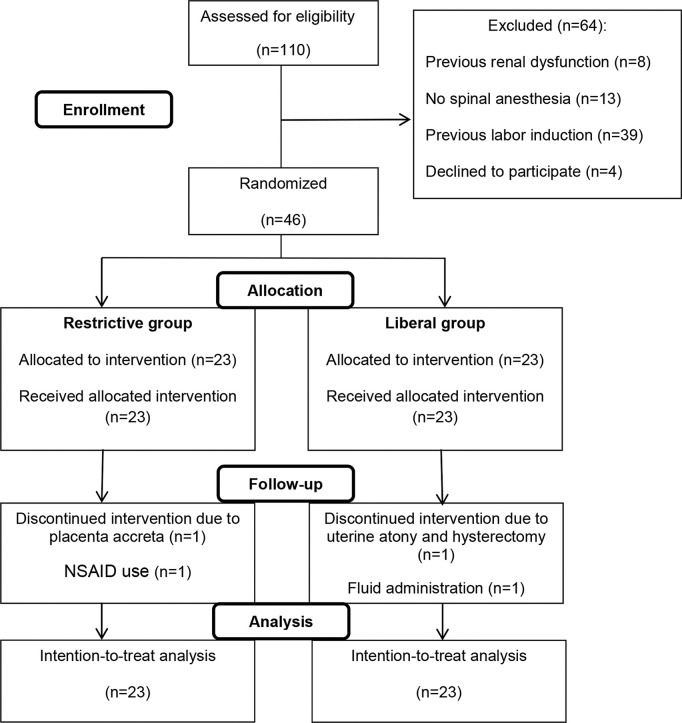
Flow chart of the study design.

Of 110 eligible patients with preeclampsia, 46 met the inclusion criteria and were enrolled in the study, 23 being allocated to the intravenous liberal regimen and 23 to the intravenous restrictive regimen. After allocation, four patients (two from each group) dropped out, although they remained in the final (intention-to-treat) analyses (Figure 1). The baseline characteristics were similar between the two groups (Table 1).

Intraoperative fluid administration, urine output, and cumulative fluid administration during the first eight postoperative hours are shown in Table 2.

### Primary outcome

The rate of postoperative AKI was 43.5% in the liberal fluid group and 43.5% in the restrictive fluid group (*p*=1.0). AKIN graduation (stage 1, 2 and 3) had no statistical difference (Table 3).

Serum creatinine was significantly higher on PO1 and PO2 than at baseline in both groups (*p*<0.05). In the restrictive group, serum creatinine was lower on PO2 than on PO1 (*p*<0.05).

### Secondary outcomes

In both groups, cystatin C was significantly lower on PO2 than on PO1 (*p*<0.05). In the restrictive group, the level of NGAL was significantly higher on PO1 than at baseline (*p*<0.05), although it was significantly lower on PO2 than on PO1 (*p*<0.05). There was no significant change in NGAL in the liberal group.

In both groups, the MDRD-estimated GFR was significantly lower on PO1 and PO2 than at baseline (*p*<0.05), whereas the creatinine/cystatin C-estimated GFR was significantly lower on PO1 than at baseline (*p*<0.05) and significantly higher on PO2 than on PO1 (*p*<0.05).

On PO1 and PO2, proteinuria was significantly lower in comparison to the baseline value in both groups (*p*<0.05). The platelet count was significantly higher on PO2 compared to PO1 in both groups (*p*<0.05).

### Post-hoc analysis

The dose of metaraminol required in the intraoperative period was significantly higher in the restrictive group. The median dose of metaraminol used in the restrictive group was 1.2 (0.9-2.2) mg *versus* 0.8 (0.6-1.4) mg in the liberal group (*p*=0.039).

The two groups did not differ in terms of the use of vasodilators, hemodialysis requirement, need for supplemental oxygen, ICU admission rate, length of hospital stay, neonatal Apgar scores, or the results of the analysis of umbilical cord blood gases (Table S1).

## DISCUSSION

In this monocentric randomized controlled pilot trial including patients with severe preeclampsia, a restrictive fluid regimen during cesarean section was not associated with increased postoperative AKI.

Intravenous fluid therapy during and after surgery restores and maintains body water, electrolytes, and organ perfusion to achieve homeostasis (17). The optimal fluid regimen in patients during cesarian section and preeclampsia is under debate (18). To our knowledge, this is the first randomized controlled trial evaluating the effects of liberal or restrictive fluid regimen during surgery on postoperative AKI in patients with severe eclampsia.

Recent clinical practice guidelines for preeclampsia recommend restricting fluid administration to ≤80 ml/h in the peripartum period to prevent pulmonary edema and other complications of fluid overload (19,20). In the United Kingdom Amniotic Fluid Embolism Register, the recommended urine output tolerance is 20 ml/h in the first 8 hours after delivery (19). In the pregnancy hypertension guidelines employed in British Columbia, the implementation of a new restrictive protocol was reported to have no effect on the relative risk of developing AKI (20).

In our study, the definition of the amount of volume administered in the restrictive group was based on experience of intraoperative hydration in pregnant women with severe heart disease, in whom aliquots of 250 mL to 300 mL of crystalloid solution were well tolerated and without repercussions (21,22). The amount administered in the liberal group was based on the institutional routine. This quantitative of fluids was considered safe since it was smaller than the volume found by Thornton et al. in a study that evaluated the amount of intrapartum fluids and the occurrence of acute edema in pregnancy (23).

Critical patients in intensive care unit submitted to restrictive fluid regimes have not been associated with worsening of their renal function (24-26). In a randomized trial comparing a liberal and a conservative strategy of fluid management in acute lung injury, the lung function was improved and the duration of mechanical ventilation and intensive care stay was reduced by the conservative strategy. The conservative strategy did not increase non pulmonary-organ failures, such as renal function (24).

Fluid overload can have an adverse effect on encapsulated organs, such as the kidneys, because it increases venous pressure and promotes interstitial edema, thus having a direct impact on kidney function (27,28). In addition, excessive fluid resuscitation is a risk factor for the development of abdominal hypertension and abdominal compartment syndrome, which can further impair renal perfusion (29). The development of fluid overload can complicate the diagnosis of AKI when that diagnosis is based on a change in serum creatinine. Serum creatinine is known to distribute into the intracellular and extracellular fluid compartments (30).

Despite the previous publications about the benefits of restrictive therapies, a recent trial with patients undergoing major abdominal surgery (Relief Trial) showed that restrictive compared to liberal fluid regimen was associated with increased AKI (31). An important data is that colloids were administrated during the perioperative period in more patients from the restrictive group than the liberal group in Relief Trial (31).

In the present study, patients in both groups showed a worsening in serum creatinine and the MDRD-estimated GFR on postoperative days, without a return to baseline. This finding suggests that the patients are still at risk for postoperative AKI even after delivery. Further, differently from previous studies (24,31), we defined *a priori* acute kidney injury using recent proposed scores and additionally, renal biomarkers have been evaluated before and after surgery. Cystatin C was significantly lower on second postoperative days suggesting an improvement in renal function in both groups. The recovery of renal function was evidenced by the fact that the creatinine/cystatin C-estimated GFR was decreased on the first postoperative day, but recovered on the second postoperative day in both groups. Our assessment of NGAL in the restrictive group indicated worsening of renal function in the immediate postoperative period, followed by improvement on the second postoperative day. A combined evaluation of these new biomarkers of renal injury suggested that the renal function of severe preeclampsia patients worsens on the first day after cesarean section, improving (returning to preoperative values) by the second day. Knowing that creatinine is a late marker, it cannot detect an improvement in renal function. A reduction in proteinuria, which is also indicative of recovery from renal injury was seen in both groups.

Cystatin C has been proposed as an alternative marker for estimating the GFR in native kidney disease (16,32). A composite equation based on serum cystatin C, serum creatinine, age, and gender has been shown to be superior to separate creatinine- and cystatin C-based equations (33). The serum concentration of NGAL has been correlated with the presence and severity of preeclampsia in several studies (10,34). The generalized endothelial injury associated with preeclampsia could upregulate circulating NGAL levels.

The occurrence of AKI in our study was higher than previous retrospective and observational studies. Huang et al. (5) found 16.8% women with pre-eclampsia/eclampsia and 60% women with HELLP syndrome complicated with AKI. Eswarappa et al. (35) found only 8% of AKIN in a retrospective review of 99 cases of postpartum AKI. However, a population-based study in Canada (3) demonstrated an important increase of AKI among patients with hypertensive disorders (adjusted increase 95%), even higher in women with gestational hypertension having significant proteinuria (adjusted increase 171%). It is important to point out that our study was restricted to a group of severe preeclampsia, which is the main reason for the higher number of AKI cases. Moreover, our study had included patients prospectively and thus did not suffer potential methodological issues related to the retrospective cohort analysis. The other studies evaluated the AKI incidence in the entire population of pregnant women or pregnant women with hypertensive disorders of all types, and not only severe preeclampsia.

In the post-hoc analyses, the dose of metaraminol required during cesarean section was higher in the restrictive fluid regimen group. Recent studies indicate that the use of crystalloids is of little or no benefit in counteracting hypotension and reducing the vasopressor dose required during cesarean section (36). Kee et al. found that infusion of 20 ml/kg of lactated Ringer’s does not change the total dose of metaraminol used during cesarean section under spinal anesthesia and also does not affect maternal or neonatal outcomes (37).

Our study has some limitations to be addressed. First, this is a pilot trial which must be confirmed in larger sample size. Second, urine output was not measured in the postoperative period because the catheter is removed early at our facility according to local guidelines. Therefore, 24-hour urine collection for the calculation of the creatinine clearance to estimate the GFR was not performed. Third, the blood pressure during the entire surgery was not collected, despite the restricted pressure control during cesarean section. Forth, there was no late follow-up of patients to assess the progression of the kidney dysfunction.

Despite these limitations, the strength of the paper is that of being the first randomized trial evaluating liberal *versus* restrictive fluid management in patients with severe preeclampsia and have potential clinical impact.

## CONCLUSIONS

In patients with severe preeclampsia, a restrictive fluid regimen during cesarean section was not associated with increased postoperative AKI. Additional studies are needed in order to identify the best fluid therapy in this patient population.

## AUTHOR CONTRIBUTIONS

Silva WA, Varela CVA, Francisco RPV, Torres MLA, Carmona MJC, Bliacheriene F and Malbouisson LMS contributed substantially to the conception, design, and execution of this work. Pinheiro AM and Scherer PC contributed substantially to the acquisition of the data. Andrade LC and Pelosi P analyzed the data and contributed in writing the manuscript. All authors read, contributed to important intellectual content, and approved the final draft of the manuscript prior to submission.

## Figures and Tables

**Table 1:** Demographic and clinical characteristics of patients at baseline.

Characteristics	Restrictive Fluid(n=23)	Liberal Fluid(n=23)	*p*-value
Age (years), mean±SD	33±5	31±8	0.536^a^
Body mass index (kg/m^2^), median (IQR)	30.1 (28.0-33.7)	34.1 (26.9-38.0)	0.325^b^
Gestational age (weeks), median (IQR)	36.7 (30.1-37.4)	36.9 (35.3-37.0)	0.581^b^
Twin pregnancy, n (%)	3 (14.3)	2 (12.15)	1.0^c^
Chronic hypertension, n (%)	9 (39.1)	11 (47.8)	0.552^d^
Previous preeclampsia, n (%)	11 (47.8)	9 (39.1)	0.552^d^
Diagnostic criteria for severe preeclampsia, n (%)			
Persistent high blood pressure	23 (100)	23 (100)	1.0^d^
Thrombocytopenia	0	1 (4.3)	1.0^d^
Impaired liver function	0	1 (4.3)	1.0^d^
Right upper quadrant or epigastric pain	4 (17.4)	6 (26)	0.47^d^
Pulmonary edema	0	0	1.0^d^
Cerebral or visual disturbances	9 (39.1)	8 (34.7)	0.689^d^
Antihypertensive drugs used before hospital admission, n (%)			
Methyldopa alone	9 (39.1)	7 (30.4)	0.6^d^
Methyldopa and pindolol	2 (8.6)	2 (8.6)	1.0^d^
Methyldopa and amlodipine	1 (4.3)	0	1.0^d^
Methyldopa, pindolol, and amlodipine	1 (4.3)	2 (8.6)	1.0^d^
Pindolol alone	2 (8.6)	4 (17.3)	0.381^d^
None	8 (35.1)	8 (35.1)	1.0^d^
Diabetes mellitus, n (%)	5 (21.7)	9 (39.1)	0.2^d^
Anesthesia and surgical time (min), median (IQR)	120 (110-135)	120 (120-140)	0.563^b^
Blood pressure immediately before spinal anesthesia			
Systolic pressure (mmHg), mean±SD	170±20	161±17	0.148^a^
Diastolic pressure (mmHg), mean±SD	105±15	100±11	0.228^a^

IQR: interquartile range; SD: Standard deviation.

a t-Student test;

b Mann-Whitney test;

c Fisher’s exact test;

d chi-square test.

**Table 2:** Intraoperative and postoperative fluid balance.

Variable	Restrictive Fluid(n=23)	Liberal Fluid(n=23)	*p*-value
**During surgery**			
Intraoperative fluid administration (ml), mean±SD	272±26	1533±34	<0.001^a^
Intraoperative urine output (ml/h), median (IQR)	80 (37-110)	116 (69-191)	0.032 b
**During the first 8 postoperative hours**			
Cumulative fluid administration (ml), median±SD	772±26	2033±35	<0.001^a^

IQR: interquartile range; SD: Standard deviation;

a t-Student test

b Mann-Whitney test.

**Table 3:** Primary and secondary outcomes.

Variable	Time point	Restrictive Fluid(n=23)	Liberal Fluid(n=23)	*p*-Value
**Primary outcome**				
AKIN stage≥1, n (%)	-	10 (43.5)	10 (43.5)	1.0^a^
AKIN graduation, n (%)				
1	-	6 (26.1)	7 (30.4)	
2	-	4 (17.4)	2 (8.7)	0.540^b^
3	-	0	1 (4.3)	
Creatinine (mg/dl), median (IQR)	Preoperative	0.73 (0.55-0.83)	0.69 (0.55-0.83)	0.879^c^
	Postoperative day 1	0.93 (0.76-1.25) † d	0.99 (0.80-1.22) † d	0.704^c^
	Postoperative day 2	0.84 (0.69-1.08) † ‡ d	0.96 (0.80-1.09) † d	0.316^c^
**Secondary outcomes**				
Cystatin C (mg/L), mean±SD	Preoperative	1.99±0.61	1.73±0.6	0.627^e^
	Postoperative day 1	2.03±0.66	1.93±0.8	
	Postoperative day 2	1.72±0.43‡	1.64±0.5‡	
NGAL (mg/L), median (IQR)	Preoperative	0.15 (0.13-0.22)	0.16 (0.10-0.20)	0.448^c^
	Postoperative day 1	0.21 (0.16-0.29) † d	0.18 (0.13-0.26)	0.255^c^
	Postoperative day 2	0.15 (0.11-0.22)‡ d	0.15 (0.10-0.24)	0.945^c^
GFR (ml/min/1.73 m^2^), mean ± SD	Preoperative	109.56±51.88	102.43±27.72	
MDRD*	Postoperative day 1	69.72±25.68†	68.62±22.74†	0.800^e^
	Postoperative day 2	78.56±23.34†	71.24±26.67†	
Serum creatinine/cystatin C#	Preoperative	56.87±18.07	69.73±22.45	
	Postoperative day 1	48.67±23.06†	55.27±17.06†	0.150^e^
	Postoperative day 2	58.53±20.13‡	58.67±21.95‡	
Proteinuria (g/L), median (IQR)	Preoperative	2.63 (0.80-9.60)	0.46 (0.32-1.71)	0.062^c^
	Postoperative day 1	0.40 (0.28-0.58) † d	0.15 (0.10-0.88) † d	0.208^c^
	Postoperative day 2	0.45 (0.10-2.23) † d	0.20 (0.10-0.48) † d	0.257^c^
Platelet count (cells × 10^3^/mm^3^), mean±SD	Preoperative	207.94±71.37	194.40±77.86	
	Postoperative day 1	201.11±61.68	183.25±83.23	0.390^e^
	Postoperative day 2	221.82±62.92‡	192.85±80.69‡	

IQR: interquartile range; SD: Standard deviation; NGAL: neutrophil gelatinase-associated lipocalin; GFR: glomerular filtration rate; MDRD: Modification of Diet in Renal Disease.

a chi-squared;

b Mann-Whitney test;

c Kruskal-Wallis;

d Friedman test;

e repeated-measures analysis of variance;

† *p*<0.05 versus preoperative;

‡ *P*<0.05 versus postoperative day 1.

* Calculated as 175 × creatinine^−1.154^ × age^−0.203^ × 0.742 × 1.21 (if black).

# Calculated on the basis of serum creatinine (Scr, in mg/dl) and serum cystatin C (Scys, in mg/L), as follows:

If Scr≤0.7 and Scys ≤ 0.8—130 × (Scr/0.7)^−0.248^ × (Scys/0.8)^−0.375^ × 0.995^Age^ [× 1.08 if black].

If Scr≤0.7 and Scys > 0.8—130 × (Scr/0.7)^−0.248^ × (Scys/0.8)^−0.711^ × 0.995^Age^ [× 1.08 if black].

If Scr > 0.7 and Scys ≤ 0.8—130 × (Scr/0.7)^−0.601^ × (Scys/0.8)^−0.375^ × 0.995^Age^ [× 1.08 if black].

If Scr > 0.7 and Scys > 0.8—130 × (Scr/0.7)^−0.601^ × (Scys/0.8)^−0.711^ × 0.995^Age^ [× 1.08 if black].

**Table:** Intraoperative and postoperative variables in patients with preeclampsia, by the type of fluid therapy applied during cesarean section: post-hoc analyses.

Variable	Restrictive Fluid(n=23)	Liberal Fluid(n=23)	*p*-value
Intraoperative medication			
Vasodilator, n (%)	1 (4.3)	2 (8.7)	1.0^a^
Vasopressor, n (%)	18 (78.3)	15 (65.2)	0.326^b^
Metaraminol (mg), median (IQR)	1.2 (0.9–2.2)	0.8 (0.6–1.4)	0.039^c^
Hemodialysis, n (%)	0	1 (4.3)	1.0^a^
Supplemental oxygen use, n (%)	7 (30.4)	6 (33.3)	0.843^b^
ICU admission, n (%)	0	2* (8.7)	0.489^a^
Time to discharge after surgery (days), median (IQR)	3 (3–5)	3 (3–3)	0.196^c^
Apgar score, median (IQR)			
At 1 minute	8 (7–9)	8 (7–8)	0.663^c^
At 5 minutes	9 (8–10)	9 (9–9)	0.621^c^
Umbilical cord blood gases, mean±SD			
pH	7.23±0.66	7.22±0.11	0.647^d^
Bicarbonate (mEq/ml)	23.71±4.07	22.13±2.29	0.190^d^
Base excess	−5.71±2.05	−6.52±3.23	0.414^d^

a Fisher’s exact test;

b chi-square test;

c Mann–Whitney test;

d t-Student test.

* one case of uterine atony and one case of persistent oliguria and neurological symptoms.

IQR: interquartile range; AKIN: Acute Kidney Injury Network; ICU: intensive care unit.
